# A feasibility study of objective outcome measures used in clinical trials of freezing of gait

**DOI:** 10.1186/s40814-022-01092-2

**Published:** 2022-07-04

**Authors:** Gonzalo J. Revuelta, Aaron Embry, Jordan J. Elm, Shonna Jenkins, Philip Lee, Steve Kautz

**Affiliations:** 1 Movement Disorders Division, Department of Neurology, College of Medicine, Medical University of South Carolina, 208B Rutledge Avenue, MSC 108, Charleston, SC 29425 USA; 2 Ralph H. Johnson VA Medical Center, Charleston, SC USA; 3 Center for Rehabilitation Research in Neurological Conditions, Department of Health Sciences and Research, College of Health Professions, Medical University of South Carolina, Charleston, SC USA; 4 Department of Public Health Sciences, College of Medicine, Medical University of South Carolina, Charleston, SC USA; 5 Department of Neurology, Virginia Commonwealth University, Richmond, VA USA

**Keywords:** Parkinson’s disease, Freezing of gait, Severity, Surrogate markers, Outcome measures

## Abstract

**Background:**

Freezing of gait (FOG) is notoriously difficult to quantify, which has led to the use of multiple markers as outcomes for clinical trials. The instrumented timed up and go (TUG) and the many parameters that can be derived from it are commonly used as objective markers of FOG severity in clinical trials; however, it is unknown if they represent actual FOG severity.

**Objective:**

To determine the specificity and responsiveness of objective surrogate markers of FOG severity commonly utilized in FOG studies.

**Methods:**

Study design: We compared the specificity and responsiveness of commonly used markers in FOG clinical trials. Markers compared included velocity, step/stride length, step/stride length variability, TUG, and turn duration. Data was collected in four conditions (ON and OFF dopaminergic drugs, with and without a dual task). Unified Parkinson’s Disease Rating Scale (UPDRS) was administered in the ON and OFF states.

**Results:**

Thirty-three subjects were recruited (17 PD subjects without FOG (PD-control) and 16 subjects with PD and dopa-responsive FOG PD-FOG). The UPDRS motor scores were 24.9 for the PD-control group in the ON state, 24.8 for the FOG group in the ON state, and 42.4 for the FOG group in the OFF state. Significant mean differences between the ON and OFF conditions were observed with all surrogate markers (*p* < 0.01). However, only dual task turn duration and step variability showed trends toward significance when comparing PD-control and ON-FOG (*p* = 0.08). Test–retest reliability was high (ICC > 0.90) for all markers except standard deviations. Step length variability was the only marker to show an area under the ROC curve analysis > 0.70 comparing ON-FOG vs. PD-control.

**Conclusions:**

Multiple candidate surrogate markers for FOG severity showed responsiveness to levodopa challenge; however, most were not specific for FOG severity.

## Background

Freezing of gait (FOG) is a debilitating condition occurring in the majority of patients with Parkinson’s disease (PD) [1–3], for which there is no effective therapy. It is defined as the episodic inability to walk, often triggered by environmental factors [4]. A major barrier toward therapeutic development in FOG is the lack of validated, objective outcome measures of FOG severity [5]. Measures like the Freezing of Gait Questionnaire [6] (FOG-Q) are limited by their subjective nature and cannot be repeated in one session since they are meant to be retrospective over a period of 1 month. The new FOG-Q has recently been found to be unreliable and not responsive to small effect sizes [7]. Measures that rely on capturing a FOG episode in the laboratory (direct measures) [8–12] are limited by the inherent variability of each episode; therefore, a captured episode may not be representative of overall FOG severity. Furthermore, approaches to reliably trigger an episode have not been established. Long-term continuous monitoring approaches [12, 13] are ideal since they capture FOG severity over a period of days or weeks accounting for variability of individual episodes; however, they cannot be repeated in one session (since they must be administered over a long term) and require the analysis of large amounts of data.

Surrogate markers of FOG severity present an option for therapeutic trials, since they are objective assessments, easy to administer, can be administered multiple times in one session, and do not depend on triggering an episode of FOG; however, their specificity for FOG severity has not been determined. This type of marker is particularly useful for dose-finding studies and determining immediate effects of therapeutic interventions, e.g., neuromodulation therapies which require testing of multiple variables to optimize. For these reasons, clinical trials of therapies for FOG have utilized multiple candidate surrogate markers including instrumented timed up and go (TUG), turn duration, velocity, dual task interference, and step length variability [14–17]. However, it is not clear which (if any) of these markers best represent FOG severity, and if they are responsive to the interventions that are being tested, impairing our ability to interpret these studies and providing little guidance for future study design.

We selected markers that have been commonly utilized as surrogate markers of FOG severity in previous studies (velocity, step length, step length variability, dual task interference [13, 14, 17, 18], and turn duration [19–21]). To determine the feasibility of objective markers as outcomes in future clinical trials aiming to improve FOG severity, we defined two feasibility objectives: (1) *specificity for FOG* and (2) *responsiveness* to intervention. Given that these markers are indirect measures of gait, specificity is of particular interest. To determine the specificity of each marker for FOG, we selected a group of patients with FOG, and a control group of PD patients without FOG (that had otherwise similar motor severity as the PD-FOG group) and tested the ability of each marker to differentiate between groups. To determine responsiveness, we ensured each of the FOG patients selected had a clear response to dopaminergic medications (dopa-response) and compared the ability of each marker to differentiate between the OFF and ON medication state.

## Methods

### Subjects

Subjects (ages 18–80) who met UK Brain Bank criteria for idiopathic PD (Hoehn and Yahr stage 2–4 [22]) were recruited from the Medical University of South Carolina Movement Disorder Clinics. Referrals were made by clinic providers and patients were then contacted by research staff and provided with information of the study. Interested potential subjects were brought into the Murray Center for Research in Parkinson’s Disease and Related Diseases for a screening visit and were consented by study staff.

### Inclusion and exclusion criteria

Subjects with a score of 0 in question 1 of the new freezing of gait questionnaire [23] (nFOGQ) and item 14 of the UPDRS part 2 were enrolled into the PD-control group. Subjects with a score of 1 in question 1 of the nFOGQ were enrolled in the FOG group. To ensure subjects in the FOG group had dopa-responsive FOG, an improvement of at least 1 point on item 14 of the Unified Parkinson’s Disease Rating Scale (UPDRS) from the OFF to the ON state was required. In addition, each subject was observed to have FOG at screening and confirmed through multiple comprehensive clinical evaluations by a movement disorder neurologist (GJR) in the ON and OFF states. Subjects who exhibited FOG due to any trigger (initiation, turning, upon reaching destination, or on straightaway walking) or any phenomenological subtype of FOG (akinetic, knee trembling) were included in the FOG group; therefore, all FOG subgroups [24] were included in this study. Subjects with a Mini-Mental Status Examination score of < 26, or who were unable to walk 30 feet unassisted in the OFF state, or had any other significant gait impairment (festination, or major orthopedic disturbance affecting gait) were also excluded from the study.

The Institutional Review Board of the Medical University of South Carolina approved the study (Pro00037836). All participants provided written informed consent to take part in this study. The datasets generated during the current study are available from the corresponding author upon request.

### Assessments

#### Clinical

All patients had full UPDRS (parts 1–4) in the practically defined ON and OFF states (OFF: 12 h off all dopaminergic agents, and ON: at least 30 min after taking dopaminergic agents), and nFOGQ, performed by a movement disorder neurologist (GJR).

#### Description of gait assessment

Spatiotemporal parameters were obtained by a research physical therapist (AE) from the GAITRite® (CIR Systems, Franklin NY) electronic walkway in the ON and OFF states with and without a dual cognitive task. The dual tasks alternated between serial 7’s and every other letter of the alphabet. Performance on the dual task was monitored to ensure adequate effort as reported in prior studies [25]. Spatiotemporal data was collected and averaged from four passes over the GAITRite® walkway (two trials). Specifically, they were asked to stand up, walk over the GAITRite® walkway, step off the GAITRite® onto the M^2^ walkway, turn around a cone set at the center of the M^2^ (54 inches to the center of the cone from the leading edge of the M2/GAITRite® interface), and walk back to the chair. The M^2^ walkway is a square digital walkway placed at the end of the GaitRite® walkway designed to capture the turn. The instructions for the walking task were identical to what is commonplace during the TUG [26]. The turn was 180° and the diameter of the turn was only limited to the 48″ width or lateral boundaries of the M^2^ walkway. The turn was performed by each subject in their preferred direction. Participants were not required to pre-select their direction of turn and were not mandated to turn in either or both directions.

#### Quantitative data generated from gait assessment

This protocol yielded two walking periods or passes on the GAITRite® per trial and one turn duration per trial. Two trials (4 passes) were completed in each condition (two trials ON dopaminergic medication, single task; two trials ON dopaminergic medication, dual task; two trials OFF dopaminergic medication, single task; and two trials OFF dopaminergic medication, dual task). The average and standard deviation (SD) was estimated for each side (left and right) for a total of four passes in each condition (each trial producing two passes on the GAITRite®, one departing and another returning to the chair). Step length was not corrected for leg length. Step and stride length coefficient of variability (CV) were calculated from the standard deviation of each parameter (again a total of 4 trials on the GAITRite® were used to calculate CV) as previously described [27]. The turn task (mean time to turn) was calculated as the difference between the moment the individual stepped off the end of the GAITRite® and onto the M2 walkway to the time of the end of the final foot fall leaving the M2 and returning to the GAITRite®. The distance from the end of the GAITRite® to the cone was kept constant for all participants. The difference for each spatiotemporal parameter with and without a concurrent cognitive task was calculated and labeled dual task interference (e.g., the measured step length without a dual task was subtracted from the measured step length with a dual task to generate step length dual task interference). If subjects experienced a freezing episode during a walking trial, accurate spatiotemporal data could not always be obtained. For those trials, manual step identification was attempted to include as many steps as possible in each trial.

Timed data (TUG and turn duration) included the occurrence of FOG episodes when they occurred. This protocol is not designed to precipitate FOG episodes or to directly measure the duration or severity of an individual episode, but rather to describe a marker’s properties to *indirectly* function as a *surrogate* of FOG severity.

### Statistical analysis

Raw spatiotemporal data was removed from the GAITRite®, into a spreadsheet for calculation of the surrogate markers of interest. Clinical data was entered into paper forms. All data was then uploaded into a RedCap database for statistical analysis. Turn duration under the dual task condition was pre-specified as the primary parameter of interest as it had been utilized effectively in a previous clinical trial for FOG (without the dual task component) [14].

#### Sample size estimation

Sample size was estimated based on the ability for turn duration to distinguish between severity groups. A prior study [14] found the mean duration turn task was 31s for the PD patients with FOG versus 2.7s for PD patients without FOG (SD = 25). Assuming a similar difference in groups and standard deviation when assessed under dual task, a two-sample *t*-test has 85% power when there are *n*=15 patients in each group with two-sided alpha=0.05.

Test–retest reliability was calculated for each spatiotemporal parameter for the 3–4 trials on a single visit using the intraclass correlation coefficient (ICC) reliability for the mean of *k* ratings (SAS %INTRACC macro). For each spatiotemporal parameter, the Wilcoxon rank sum test was used to compare group differences in FOG patients to PD-control. Similarly, the Wilcoxon signed-rank test was used to determine whether there were differences in response to dopaminergic medications (dopa-response) within FOG patients (tested under the ON and OFF condition, respectively). The statistical significance level was set at alpha=0.05 for all comparisons. These analyses are purely to demonstrate the measurement properties of the spatiotemporal parameters by examining the extent to which the means differ in the expected fashion using groups that are known to be different (ON-FOG, OFF-FOG, and PD-control). Results will be presented with 95% confidence intervals.

Area under the receiver operating characteristic curve (AUC) analysis was performed as a measure of responsiveness (or the ability to distinguish one group from another) for each spatiotemporal parameter. This was done by fitting a series of logistic models of PD-control versus PD-FOG as the response modeled with a separate model for each dopa-response condition (ON/OFF). Similarly, a logistic model with a random effect for subject was fit with the ON/OFF condition as the response (PROC GLIMMIX). AUC values of 0.70 or higher are generally considered adequate to demonstrate that a measure is able to distinguish one group from another [28].

## Results

### Demographic and clinical descriptive data

The mean (SD) PD-control (no FOG) group (*n* = 17) was 67.3 (5.4) years of age, 5.2 (3.7) years of disease duration, with 6 females, 15 whites, one African American, and one of other race/ethnicity. The mean (SD) PD-FOG group (*n* = 16) was 64.3 (5.7) years of age, 10.2 (4.6) years of disease duration, with 5 females, all whites.

The mean UPDRS, part III (motor) scores were 24.8 (10.4) for the PD-control group in the ON condition, 24.2 (9.1) for the FOG group in the ON condition, and 42.4 (8.6) for the FOG group in the OFF condition. The UPDRS part II, item 14 FOG scores (a subjective measure of FOG severity) were 0 (0) for the PD-control group, 0.8 (0.7) for the FOG group in the ON condition, and 2.6 (0.6) for the FOG group in the OFF condition (severe FOG severity level). The mean nFOGQ score was 17.8 (5.5) in the FOG group and 0 in the PD-control group.

### Test–retest reliability

Given that an AUC of > 0.70 is generally considered to be adequate [28], test–retest reliability of the spatiotemporal parameter under a single type of condition (i.e., SINGLE or DUAL) was high (ICC > 0.90) for all measures, except the standard deviation (SD) measures (e.g., step length standard deviation left, etc.). ICC was poor (< 0.50) for the SD measures under the SINGLE condition and fair under the dual task condition for the PD patients with FOG in the ON state, PD patients with FOG in the OFF state, and the PD-control subjects. See Table 1.

**Table 1:** Test–retest reliability for the gait parameters in PD-controls and PD-FOG

	**PD-control**		**PD-FOG**			
	**Single**	**Dual**	**ON**		**OFF**	
			**Single**	**Dual**	**Single**	**Dual**
	**ICC**					
Velocity (m/s)	0.98	0.98	0.97	0.96	0.97	0.98
Step length, R (cm)	0.99	0.99	0.99	0.98	0.99	0.98
Step length, L (cm)	0.99	0.99	0.99	0.98	0.99	0.97
Step length SD, R (cm)	0.33	0.57	0.25	0.59	0.62	0.95
Step length SD, L (cm)	0.23	0.66	0.68	0.68	0.68	0.86
Stride length, R (cm)	0.99	0.99	0.99	0.98	0.99	0.99
Stride length, L (cm)	0.996	0.99	0.99	0.98	0.99	0.98
Stride length SD, R (cm)	0.29	0.53	0.16	0.77	0.68	0.97
Stride length SD, L (cm)	0.36	0.71	0.53	0.69	0.70	0.92

### Comparison of surrogate markers

The group means (or medians) were different for all spatiotemporal measures, with and without a dual task, between the PD-control versus PD-FOG-OFF groups and for the PD-FOG-ON versus PD-FOG-OFF condition within the FOG group. However, no differences in the means/medians were detected between the PD-FOG-ON and PD-control groups, with only trends for dual task step CV and dual task turn duration. See Table 2. The dual task interference for average step length and average stride length was significantly different between the PD-control versus PD-FOG-OFF groups, but no other group differences in the dual task interference metrics were detected.

**Table 2:** Spatiotemporal data across groups

	**PD-control (no FOG) (*n* = 17)**			**Freezers (FOG) (*n* = 16)**						***p*-value**		
				OFF			ON					
	Median (IQR)	Mean difference control-OFF 95% CI	Effect size	Median (IQR)	Mean difference control-ON 95% CI	Effect size	Median (IQR)	Mean difference OFF-ON 95% CI	Effect size	Control vs OFF	Control vs ON	ON vs OFF (paired)
Dual task												
Velocity (m/s)	108.9 (95.7, 120.3)	39.9 (21.0, 58.8)	1.53	74.0 (52.8, 89.9)	12.1 (−3.1, 27.2)	0.57	95.0 (85.2, 114.3)	−28.2 (−38.9, −17.5)	1.46	<0.001	0.19	<0.001
Step length (R and L) (cm)	59.0 (52.4, 68.5)	20.9 (10.8, 31.1)	1.49	39.5 (29.6, 50.1)	5.6 (−3.6, 14.9)	0.43	51.9 (48.8, 58.4)	−15.6 (−21.2, −10.0)	1.55	<0.01	0.14	<0.001
Stride length (R and L) (cm)	117.9 (102.5, 136.7)	41.3 (21.0, 61.7)	1.47	79.2 (59.2, 100.2)	10.9 (−7.7, 29.6)	0.42	104.1 (96.1, 117.7)	−31.3 (−42.6, −20.0)	1.54	<0.01	0.20	<0.001
Step CV	4.5 (3.3, 5.9)	−13.1 (−26.6, 0.3)	0.77	7.4 (5.4, 15.0)	−2.1 (−4.1, 0.0)	0.81	6.0 (5.1, 8.7)	11.0 (−2.3, 24.3)	0.50	<0.01	0.08	<0.01
Stride CV	3.2 (2.4, 5.0)	−10.8 (−21.8, 0.3)	0.77	5.3 (4.2, 13.1)	−1.4 (−3.2, 0.5)	0.59	3.9 (3.2, 5.6)	9.4 (−1.3, 20.2)	0.53	0.01	0.25	<0.01
Time to turn (s)	5.0 (4.3, 5.7)	−13.6 (−25.4, −1.8)	0.83	8.2 (6.2, 19.8)	−1.2 (−2.4, 0.0)	0.71	5.5 (5.0, 6.5)	12.4 (−0.3, 25.0)	0.54	<0.01	0.08	<0.01
Single task												
Velocity (m/s)	128.2 (114.0, 137.7)	36.3 (16.3, 56.4)	1.29	94.5 (67.4, 106.9)	12.8 (−2.8, 28.5)	0.58	111.2 (100.7, 131.8)	−23.5 (−35.0, −12.0)	1.09	<0.01	0.10	<0.001
Step length (R and L) (cm)	67.4 (58.6, 73.9)	16.2 (5.3, 27.1)	1.06	51.3 (39.1, 57.9)	4.6 (−4.9, 14.0)	0.34	61.2 (54.6, 64.8)	−11.6 (−16.0, −7.3)	1.43	<0.01	0.40	<0.001
Stride length R and L (cm)	135.3 (117.5, 148.1)	31.3 (9.6, 53.1)	1.02	103.0 (78.4, 116.1)	8.5 (−10.2, 27.1)	0.32	122.7 (109.4, 130.3)	−22.9 (−31.8, −14.0)	1.37	<0.01	0.35	<0.001
Step CV	3.9 (2.8, 4.2)	−8.1 (−16.5, 0.4)	0.76	6.4 (5.4, 8.6)	−0.8 (−2.1, 0.5)	0.46	4.0 (3.7, 4.7)	7.3 (−0.9, 15.5)	0.54	<0.01	0.33	<0.001
Stride CV	2.8 (2.1, 3.3)	−7.2 (−15.1, 0.7)	0.73	5.0 (3.7, 7.8)	−0.6 (−1.6, 0.4)	0.50	3.1 (2.7, 3.6)	6.6 (−1.3, 14.5)	0.51	<0.01	0.26	<0.001
Time to turn (s)	4.4 (3.6, 4.8)	−6.9 (−12.5, −1.3)	0.90	6.1 (4.8, 9.9)	−0.9 (−2.0, 0.3)	0.54	4.9 (4.2, 5.4)	6.0 (0.6, 11.5)	0.61	<0.01	0.10	<0.01
Interference												
Velocity (m/s)	−15.3 (−20.1, −8.9)	5.2 (−6.4, 16.9)	0.32	−14.8 (−36.9, −10.1)	−0.8 (−8.1, 6.6)	0.07	−13.2 (−24.5, −8.1)	−5.3 (−14.6, 4.1)	0.31	0.74	0.84	0.45
Step length (R and L) (cm)	−5.4 (−7.1, −3.8)	5.9 (1.3, 10.6)	0.93	−8.6 (−21.5, −6.3)	1.0 (−2.1, 4.2)	0.24	−7.2 (−10.9, −4.1)	−4.4 (−8.4, −0.3)	0.60	<0.05	0.26	0.06
Stride length R and L (cm)	−11.0 (−14.1, −8.9)	12.7 (3.8, 21.7)	1.03	−17.3 (−43.6, −12.8)	2.5 (−3.3 − 8.3)	0.30	−14.0 (−21.4, −7.1)	−9.2 (−17.6, −0.9)	0.61	<0.01	0.28	0.06
Step CV	0.7 (−0.6, 2.7)	−5.0 (−14.1, 4.0)	0.44	0.6 (−0.1, 1.6)	−1.3 (−2.7, 0.0)	0.79	2.1 (1.2, 3.2)	3.7 (−5.8, 13.2)	0.24	0.65	0.06	0.74
Stride CV	0.9 (−0.9, 1.6)	−3.6 (−10.5, 3.4)	0.41	0.7 (−0.3, 1.2)	−0.7 (−1.9, 0.5)	0.48	1.1 (0.4, 1.9)	2.8 (−4.5, 10.2)	0.23	0.94	0.35	0.95
Time to turn (s)	0.6 (0.2, 0.9)	−6.7 (−13.6, 0.2)	0.70	2.1 (0.3, 5.6)	−0.3 (−1.0, 0.3)	0.36	0.5 (0.2, 1.3)	6.3 (−1.6, 14.2)	0.44	0.08	0.58	0.17

**Fig. 1:**
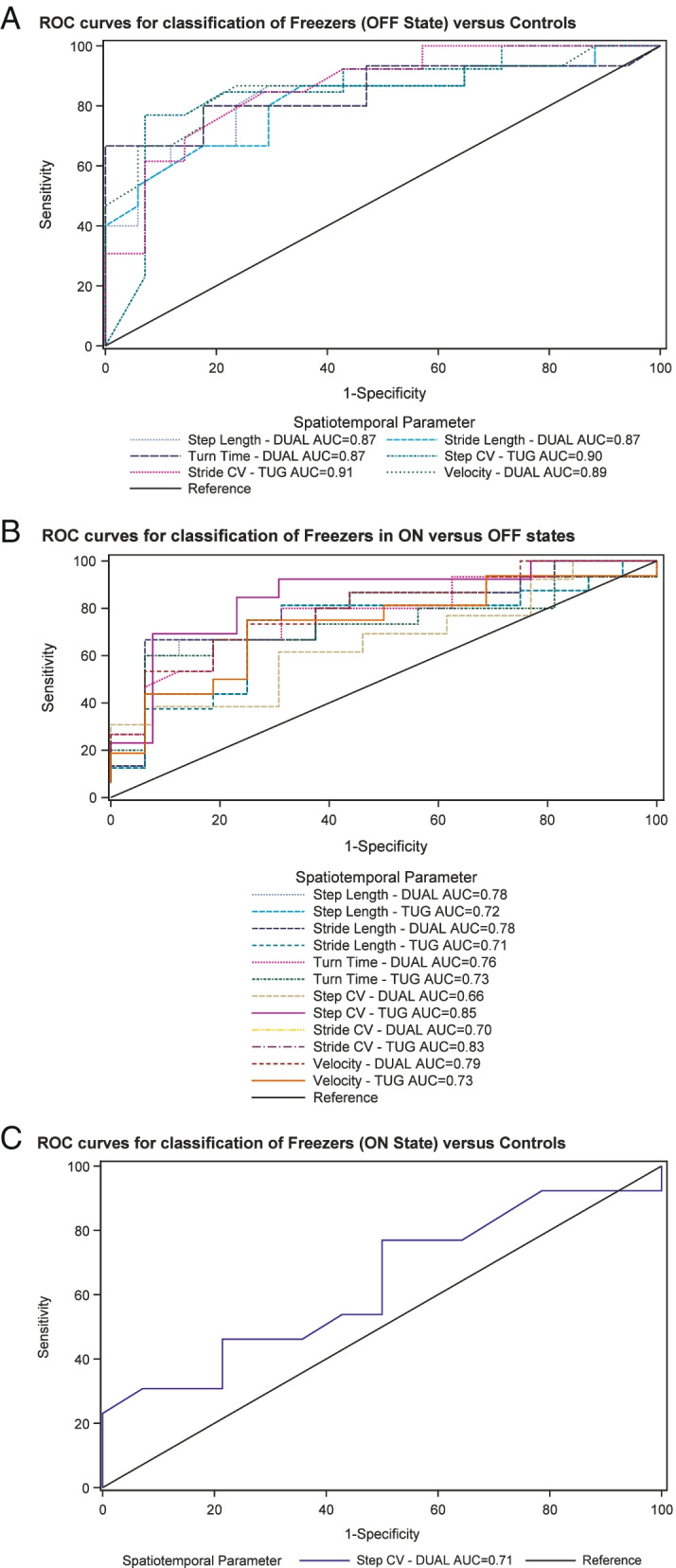
Graphical representation of the area under the receiving operating characteristics (AUC) analysis, showing sensitivity and specificity for each surrogate marker comparing PD-control (no FOG) vs. ON-FOG (**A**), PD-control vs. OFF-FOG (**B**), and ON-FOG vs. OFF-FOG (**C**)

For the area under the ROC curve (AUC) analysis, all dual task and single task spatiotemporal metrics had AUC > 0.70 when discriminating between PD-control vs. PD-FOG-OFF. Likewise, all dual task metrics and single task metrics had AUC > 0.70 when discriminating between PD patients with FOG in the ON vs. OFF condition. However, only one metric, step CV under dual task, had AUC > 0.70 when discriminating between PD-control vs. PD-FOG-ON. See Fig. 1. For the dual task interference metrics, very few had AUC greater than 0.70, namely average step length (AUC = 0.76) and average stride length (AUC = 0.79) when comparing control versus off and step CV (AUC = 0.73) when comparing control versus ON. In sum, although many markers are capable of differentiating very different groups, particularly showing responsiveness, only step CV in the dual task condition was able to differentiate groups that were very similar except for the presence of FOG, demonstrating specificity for FOG.

## Discussion

We report our findings on direct comparisons of commonly used outcome measures in FOG clinical trials. The study was designed to determine the feasibility of the use of objective markers of FOG severity in clinical trials of interventions for the treatment of FOG. The feasibility objectives include (1) the specificity of each marker for FOG and (2) the responsiveness of each marker to an intervention. In addition, we investigated whether adding a dual task or calculating dual task interference changed the biometric properties of each marker or should be considered as a separate marker. The goal of our study was to provide objective data regarding the utility of each of these markers for clinical trials or behavioral association studies in order to assist investigators in choosing the appropriate marker for the scientific question being asked. The findings of our study can inform future clinical trials investigating the effectiveness of novel interventions for FOG and can help interpret previous trials that have reported changes in these surrogate markers.

All of the surrogate markers studied were able to differentiate between ON and OFF indicating the responsiveness to levodopa challenge with and without a dual task. However, only step CV in the dual task was able to distinguish between the PD-control group and the FOG group when ON medications (AUC > 0.70). These were two very similar groups (with very similar UPDRS scores) who only differed by the fact that the FOG group had the underlying propensity for FOG behavior when in the OFF state. These findings imply that the remaining markers are not specific for FOG; however, the rigorous design of this study comparing very similar groups should be taken into account when interpreting this finding. Therefore, most of these markers may be used in clinical trials to study the magnitude of response to an intervention, however, may not to represent a change in FOG severity independently of other gait factors. Turn duration in the dual task condition showed a strong trend toward significance when comparing the ON-FOG group and the PD-control. Therefore, dual task turn duration should not be ruled out as a proxy for FOG severity in cross-sectional studies or imaging-behavioral associations investigating the relationship of a specific finding to FOG, or as an outcome in clinical trials of a therapeutic intervention. Similar markers like stride time variability have been shown to correlate with overall disease severity [27] and have also been shown to be greater in patients with PD and FOG as compared to PD alone [29, 30].

Patients with PD have been shown to have decreased automaticity of motor tasks [31] and of gait [32, 33]. Automatic gait generates effective stepping with little or no variability; therefore, it is not surprising that there is increased variability of step length in patients with FOG compared to those without FOG. Increasing cognitive load challenges automatic gait by diverting cortical control from gait. If there is a deficit in gait automaticity, greater declines in gait would be expected as a cognitive load is added. In fact, dual task interference is considered a marker for gait automaticity [34]. We interpret the finding that step CV in the dual task was the most specific marker of FOG severity to be related to a loss of automaticity of gait in FOG.

Turn duration is a very simple metric to obtain and has been utilized effectively in clinical trials for FOG in the past [35]. Our finding that adding the dual task to multiple surrogate markers improves the biometric properties of the marker informs this and future studies when selecting markers of this condition. Curtze et al. found that turning measurements were the strongest correlates of disease severity as measured by the UPDRS, in a large PD cohort with similar disease duration, although this study did not look at FOG [36]. It is important to note that although some patients may experience a FOG episode during turning (particularly the severe FOG level), this setup (using a large turning space and a cone) is designed to minimize — not precipitate — a FOG episode, and each parameter’s value is an average of at least two trials in each condition. Therefore, these results are independent of whether or not a FOG episode is triggered and differ from studies of the turn condition designed to trigger a freezing episode and then quantify each episode individually. By understanding the biometric properties of markers of FOG severity that do not depend on eliciting a FOG episode, we can remove the inherent variability of the episode, presumably allowing a more consistent and representative assessment of FOG severity. Furthermore, such a marker is inherently simple to capture and can be repeated in one session, making it ideal for same day dose-finding studies or early-stage neuromodulation clinical trials. However, this comes at the cost of specificity for FOG, for most of the parameters derived from this approach.

Study limitations include our inability to determine which condition (ON or OFF) best indicates severity, since we were comparing each marker in the ON and OFF states. However, other studies have assessed turn measurements and have found the OFF condition to be superior [36]. We were powered to determine a difference between PD-controls and PD-FOG, but not between ON and OFF FOG, or ON FOG and PD-controls. Small sample size is also a limitation and should be taken into consideration when interpreting *p*-values, especially trends. Therefore, non-significant differences or strong trends should not be discarded. Also, due to the design, we could not compare each marker’s ability to differentiate between severity levels with the nFOGQ. This is due to the fact that retrospective subjective questionnaires, when administered, provide an overall assessment of severity over a period of time (usually weeks) and cannot be administered reliably to predict severity in the ON and OFF states. There was a small difference in age between the control and FOG groups (67.2 years for the control and 64.3 years for the FOG group) and a significant difference in disease duration (5.2 years control, 10.2 years FOG group). The disease duration difference is to be expected as FOG occurs later in the disease course. Finally, this is not a validation study of any one surrogate marker, but our findings help identify the most appropriate markers to answer future scientific questions or to be used in clinical trials and should lead to future validation studies of such.

Based on the findings of this comparative study of surrogate markers of FOG severity, we conclude that (1) objective gait assessment is a feasible outcome measure in clinical trials and behavioral association studies for FOG, (2) dual task turn duration and dual task step CV are most specific for FOG of the markers compared, and (3) velocity, step/stride length, and dual task turn duration are responsive to levodopa challenge. Further validation studies of these surrogate markers are warranted for their use as outcome measures in clinical trials. As more markers become available such as continuous monitoring, novel approaches to capture FOG, or more nuanced calculations of dual task interference (e.g., dual task effect [37]), studies to validate and compare them can help guide their use in future clinical trials.

## Data Availability

The authors believe the data necessary for analysis and interpretation is provided in the manuscript; however, any further data found to be necessary can be made available by request.

## Abbreviations

PD: Parkinson’s disease
FOG: Freezing of gait
TUG: Timed up and go
UPDRS: Unified Parkinson’s Disease Rating Scale
ROC: Receiver operating characteristic
SD: Standard deviation
CV: Coefficient of variability
AUC: Area under the curve
